# Adjuvant immunotherapy in resected esophageal squamous cell carcinoma: a gospel to the non-pCRs

**DOI:** 10.1038/s41392-021-00722-0

**Published:** 2021-08-23

**Authors:** Zhihui Zhang, Nan Sun, Jie He

**Affiliations:** 1 Department of Thoracic Surgery, National Cancer Center/National Clinical Research Center for Cancer/Cancer Hospital, Chinese Academy of Medical Sciences and Peking Union Medical College, Beijing, China; 2 State Key Laboratory of Molecular Oncology, National Cancer Center/National Clinical Research Center for Cancer/Cancer Hospital, Chinese Academy of Medical Sciences and Peking Union Medical College, Beijing, China

**Keywords:** Tumour immunology, Cancer, Therapeutics

A recent study^1^ published in *The New England Journal of Medicine* by Kelly RJ presented interim results from a phase 3 clinical trial (CheckMate 577), a global, randomized, double-blind, interventional trial to evaluate a novel approach of adjuvant nivolumab after neoadjuvant chemoradiotherapy (nCRT) and surgery for esophageal or gastroesophageal junction (GEJ) cancer. The work highlighted the preliminary efficacy and safety of immune checkpoint inhibitor (ICIs) treatment for patients without a pathological complete response (pCR) in adjuvant settings, hoping to extend this benefit to additional patients.

Patients included in the CheckMate-577 study were diagnosed with resected stage II or III esophageal or GEJ cancer and received nCRT. Detailed inclusion criteria include age ≥18, resected stage II or III, residual pathological disease, and histological confirmation of adenocarcinoma or squamous cell carcinoma after chemoradiotherapy and operation. The primary and secondary endpoints were disease-free survival (DFS) and overall survival (OS). In all, 794 participants were randomized and assigned to a 1-year course of adjuvant therapy of anti-PD-1 antibody nivolumab versus placebo in the proportion of 2:1 following adjustments to assure baseline comparability. The results showed that the median DFS of the nivolumab group (22.4 months, 95% CI: 16.6–34.0) was significantly improved compared with the placebo group (11.0 months, 95% CI: 8.3–14.3), and the hazard ratio for disease progression or death was 0.69 (96.4% CI: 0.56–0.86). Nivolumab demonstrated an acceptable toxicity profile, and the occurrence rate of grade 3 or 4 adverse events was 13% in the nivolumab group versus 6% in the placebo group. In addition, patients’ health-related quality of life improved similarly in both the nivolumab and placebo groups.

Importantly, the authors observed profoundly improved survival for nivolumab versus placebo, especially for patients with esophageal squamous cell carcinoma (ESCC) (HR, 0.62) compared with adenocarcinoma (EAC) (HR, 0.75).

This led us to think about the Chinese population. Here, ESCC is the predominant tumor type and accounts for more than 90% of cases. ESCC imposes a heavy disease burden and advances in its treatment have been frustratingly slow. Modest survival benefits were observed in the combined-modality therapy of nCRT followed by surgery, over surgery alone, for patients with locally advanced esophageal cancer. The effectiveness of this treatment was well-established by the CROSS-study results. It was not until the appearance of NEOCRTEC5010 that nCRT followed by surgery was recommended as a first-line therapy for ESCC in China. However, the outcomes of this combined therapy were inconsistent, and only about a third of ESCC patients achieve pCR.^2^ Failure to achieve pCR remains a major challenge related to ESCC treatment. Many patients experience treatment failure with poor outcomes and severe adverse reactions from chemoradiotherapy, which lacks a bystander tumor-killing effect. Therefore, comprehensive investigation of effective adjuvant treatments for non-pCRs after nCRT represents an urgent and unmet need. In fact, studies have tried to add more systemic chemotherapy or chemoradiotherapy postoperatively to the non-pCRs, with marginal benefit.^3^ In this study, the introduction of adjuvant nivolumab to nCRT direct caused the median DFS to *double*; this is a huge new treatment advance for non-pCR patients.

Previous studies highlighted the obvious benefits of ICIs for treating ESCC. To date, the concomitant or sequential evaluations of combination approaches of immunotherapy in pre-and/or post-surgery with chemotherapy, radiotherapy, and other immune inhibitors have shown mixed results^4^ (Table 1). Therefore, true advances are needed to shed light on current treatment options, design a timely therapeutic schedule, and identify an effective combination pattern for treating ESCCs.

**Table 1:** Summary of published clinical trials of neoadjuvant or adjuvant use of ICIs in ESCC

Trials	Setting	Phase	Patients	Therapy	Primary outcome
NCT03914443	Neoadjuvant	1	36	Nivolumab + CF or DCF, surgery	Safety
NCT02844075	Neoadjuvant and adjuvant	2	26	Pembrolizumab + taxol/carboplatin, surgery	pCR (46.1%)
UMIN000034373	Adjuvant	2	56	5-FU + cisplatin, Atezolizumab	CR rate
CheckMate 577	Adjuvant	3	230	Platinum-based chemoradiation, surgery, Nivolumab	DFS (Nivolumab vs placebo group: 22.4 months vs 11.0 months)

*ICIs* immune checkpoint inhibitors, *ESCC* esophageal squamous cell carcinoma, *CF* 5-FU, CDDP, *DCF* 5-FU, CDDP, DTX, *pCR* pathological complete response, *CR* complete response, *DFS* disease-free survival rate

CheckMate 577 is the first truly feasible and effective blueprint for a combined treatment option for patients with ESCCs. Notably, this trial only included patients classified as non-pCR, rather than all patients with locally advanced ESCC. This allowed for more personalized treatment while reducing the likelihood of overtreatment. We also noticed that another phase II clinical trial (NCT02844075) assessed the efficacy of preoperative chemoradiotherapy and pembrolizumab in neoadjuvant and adjuvant settings for patients with locally advanced ESCC.^4^ The results demonstrated the promising benefit of this combination which could improve the pCR rate. However, the method has not been widely applied or validated because of sample size and phase II trial-related limitations. This inspired us to examine the addition of ICIs throughout the whole treatment period as a superior combination for increasing the pCR rate in the preoperative stage and the mortality rate in the postoperative stage in non-pCR patients. Thus, parallel controls should be established to compare the effects of combined ICIs treatment on the pre- and postoperative statuses of non-pCR patients.

Several issues require resolution before widespread application of this approach. PD-L1 remains a widely validated and accepted biomarker for patients with ESCCs who receive anti-PD-1/PD-L1 monoclonal antibody therapy.^5^ The authors indicated that adjuvant nivolumab therapy had similar efficacy, regardless of PD-L1 expression. However, the authors failed to consider the relationship between immunotherapy benefit and PD-L1 expression stratified by different histology types. We also suggest the authors reevaluate the predictive value of PD-L1 for exclusive ESCC. Besides, while the reported DFS could partly explain the positive efficiency, constant and close follow-up is necessary to achieve durable and convincing improvements in OS.

Our team sought to reveal the association between tumor immune status and occurrence and development of ESCC. We successfully constructed the first individualized and well-validated immune signature to effectively predict ESCCs treated with nCRT. Our signature has strong prognostic accuracy and may greatly improve prognosis.^2^ Although the benefit of adjuvant nivolumab was observed in this clinical trial, most patients will not benefit from adjuvant nivolumab therapy. Thus, identifying biomarkers to effectively predict who would derive benefit from a signal or combined approach is necessary, and exploration of more contemporary biomarkers should be keep going in this study.

Progress in improving the prognosis of non-pCRs with locally advanced esophageal cancer has been arduous and challenging. The success of the CheckMate 577, achieved by ICIs therapy in an adjuvant setting, absolutely revolutionized the landscape of cancer treatment and provide a new standard of care, especially for patients with ESCC who are non-pCR status.
